# The Effect of Lower Limb Exoskeleton Alignment on Knee Rehabilitation Efficacy

**DOI:** 10.3390/healthcare10071291

**Published:** 2022-07-12

**Authors:** AmirHossein MajidiRad, Yimesker Yihun, Nils Hakansson, Allyson Mitchell

**Affiliations:** 1 Robotics Lab, Mechanical Engineering, University of North Florida, Jacksonville, FL 32224, USA; n01459239@unf.edu; 2 Robotics and Control Lab, Mechanical Engineering, Wichita State University, Wichita, KS 67260, USA; yimesker.yihun@wichita.edu; 3 Human Biomechanics Design Lab, Biomedical Engineering, Wichita State University, Wichita, KS 67260, USA; nils.hakansson@wichita.edu

**Keywords:** exoskeleton alignment, gait, knee extension and flexion, musculoskeletal modeling

## Abstract

This study focuses on a musculoskeletal analysis of human lower extremity and associated muscle forces during different rehabilitative tasks and exoskeleton alignment models. By changing the size and orientation of the impairment levels that could be caused by the misalignment of the exoskeleton and biological knee joint, muscle stress variations were observed. This indicates an increase in force such as that generated by the Vastus lateralis muscle up to 4.3% due to a 5 mm lateral offset from an anatomically healthy knee joint location. In another setting, while a subject moved the shank through a circular trajectory using an exoskeleton support, muscle strain due to misalignment was reflected at the rectus femoris with a variation of 44%, the biceps femoris large head with 32% and the gastrocnemius muscles with 31–33% variation. These results suggest that misalignment should be taken into account while using exoskeletons with certain trajectories for knee rehabilitation purposes. Based on the shortcomings of conventional physiotherapeutic tasks, the outcome of this study can be helpful in prescribing an impactful yet convenient configuration toward a safe and promising rehabilitation process. Assessment of exoskeleton alignment during rehabilitation is important to ensure user safety with a better therapy efficacy.

## 1. Introduction

The use of assistive wearable exoskeletons and robotic devices in rehabilitation and therapeutic training is prolific, which makes them a focus in medical centers and training institutes. For people with neuromuscular diseases such as amyotrophic lateral sclerosis (ALS), muscular dystrophy (MD), myopathy or stroke, these devices can help maintain and regain limb function [1]. However, a major limitation with current assistive robotic devices and wearable exoskeletons is fit with regard to proper joint alignment. Joints are one of the most delicate and complex parts of the human skeletal system due to changes in the rotational axes over the range of motion. Typically, human joints are associated with and modeled upon measured motion kinematics, muscle forces and bone contact forces [2]; however, some modeling techniques have limited medical practicality because of simplified assumptions. Furthermore, the aforementioned diseases and conditions, as well as injuries, can lead to variations/anomalies in the kinematics, muscle responses and joint reaction forces associated with anatomical joint movement and compound efforts to model joint motion. The efficacy of wearable exoskeletons and robotic devices is dependent on proper fit and alignment between anatomical and device joints. Misalignment introduced by diseases or injuries may result in pressure to the soft tissues or, in excessive cases, may cause joint dislocation. For example, the common approach is to design a revolute/hinge joint for an exoskeleton that crosses the knee joint even though the knee joint axis moves as a function of the knee joint angle [3]. An exoskeleton joint design that does not account for the complex motion of the joint(s) may cause discomfort and injury. As a result, it is important that future exoskeleton mechanisms be capable of performing complex three-dimensional motions exhibited by their corresponding anatomical joint, e.g., the knee joint [4]. Kinematic compatibility of the exoskeleton design with anthropometric characteristics of the users is still a challenge and appropriate alignment between exoskeleton and anatomical joints should be provided [5,6] through methods such as passive slip [7]. Quantitative-based performance measures have also been employed to address human–device interaction. In one approach, the relative motion between human and exoskeletons can be observed, and a human joint motion prediction protocol can be expressed in terms of angular motion of the exoskeleton during gait [8]. The same approach has been used to assess human–machine kinematics compatibility, which requires marker placement, post processing and fitting with human body models to produce precise measurements. Task-based therapeutic training has also benefited from utilizing robotics devices [9].

**Figure 1:**
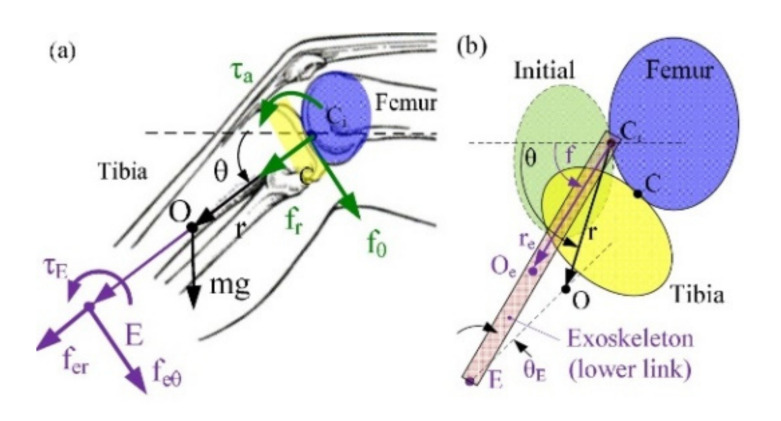
Knee rotation model developed by Wang et al. (**a**) forces, (**b**) kinematics (C_i_ pin joint, O_e_ mass center with the coordinates (r_e_, ϕ), Θ_E_ angle of misalignment between the exoskeleton and the axis of the lower leg, f_er_ and f_eθ_ forces at point E acting in er and eθ directions, τ_g_ torque due to gravitation, τ_E_ torque due to actuation [12].

As an alternative to the risks associated with exposing humans to robot counterparts, computational models of an active orthosis have been utilized to assist knee movement during human–robot interaction (HRI) with promising results [10]. Musculoskeletal simulations could help identify the lower extremity functional range of motion which is accordingly used in the optimization of the synthesis procedure. This then leads to finding suitable exoskeleton measures, segments and joints that best recreate the measured limb motions without the need of aligning a human joint to the corresponding exoskeleton joint. Due to its complex nature and susceptibility to injury, the knee joint is an appropriate candidate joint for the creation of a musculoskeletal model to study the effects of exoskeleton joint misalignment. Several studies have investigated the bio-joints and pin-based exoskeleton joints by analytical and MRI techniques [11]. Kinematics of such systems are analyzed through different motion trajectories (Figure 1) [12]. Although there are many related studies on moving the knee joint axis over various different subjects [13,14,15,16], it seems more research is required to develop a comprehensive insight through the sensitivity of exoskeleton design so as to provide a more reliable human-exoskeleton coordination. Table 1 presents a brief summary of similar studies, presenting their characteristics and limitations. It highlights the contribution of the present work and how it benefits the field through exploring new dimensions in knee rehabilitation practice.

Therefore, the objective of this study is to determine the effect of lower extremity impairment due to exoskeleton knee joint misalignment during gait and a prescribed circular motion of the shank and foot. The outcome of this analysis could identify possible complications due to utilizing exoskeleton devices in rehabilitation training. Additionally, an assessment of the severity of exoskeleton misalignment based on muscle force criterion will be presented. This can eventually lead to proposing a procedure to define suitable exoskeleton measures, segments and joints that permit the limb motions without the need of aligning an exoskeleton joint to the corresponding human joint.

## 2. Materials and Methodology

**Figure 2:**
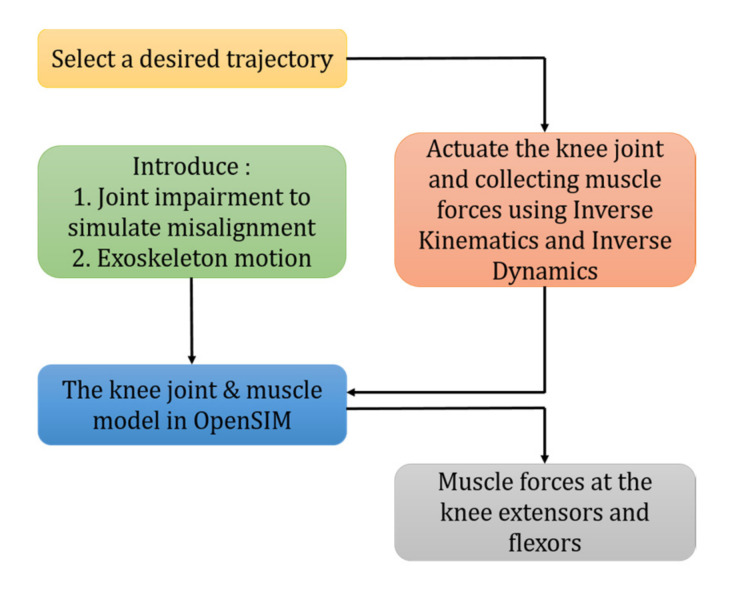
Process flowchart.

Wichita State University reviewed and approved this study under IRB4107 on 19 April 2018. This article follows the outline shown in Figure 2. OpenSIM 4.0 and the gait2392—simbody model [18], which has 23 degrees-of-freedom consisting of 92 muscles, was modified and used to implement the conditions of interest and generate simulations of the resulting motion. In all, four simulated movement patterns associated with conditions of interest were generated and analyzed. The motions and conditions were: (1) knee flexion and extension in the sagittal plane during normal gait, (2) knee flexion and extension in the sagittal plane during gait with a misaligned exoskeleton crossing the knee joint, (3) unimpaired normal right shank and foot clockwise rotation motion while seated [4], and (4) right shank and foot clockwise rotation motion while seated with a knee constraining exoskeleton. Model coordinates and skeletal alignments were modified to properly replicate the constraints for each movement.

**Figure 3:**
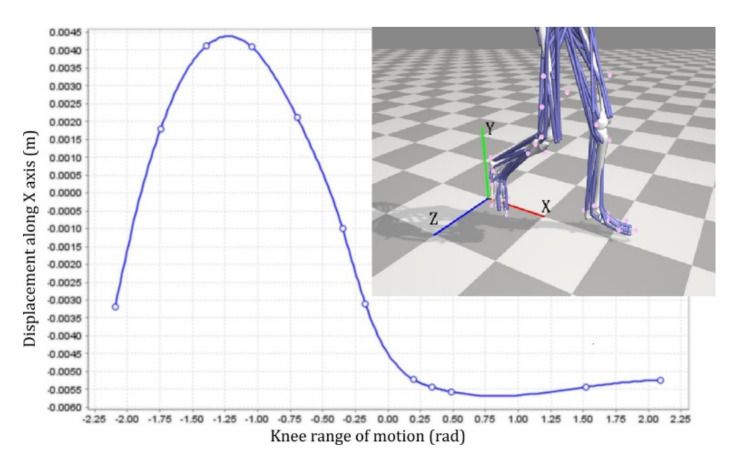
Knee movement along x-direction (i.e., sagittal plane) with respect to the knee joint angle.

**Figure 4:**
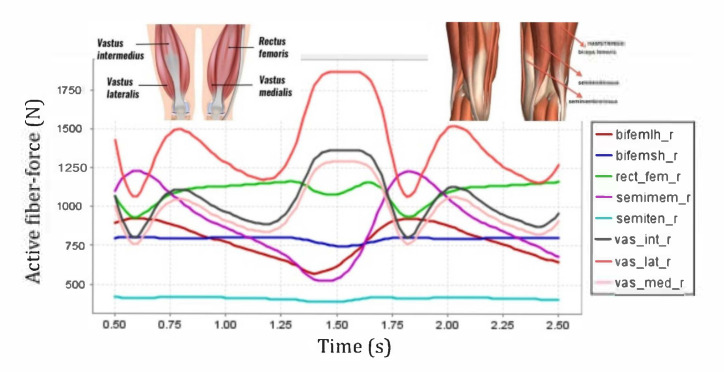
Forces of the right leg biceps femoris long head (bifemlh_r), biceps femoris short head (bifemsh_r), rectus femoris (rect_fem_r), semimembranosus (semimem_r), semitendinosus (se-miten_r), vastus intermedius (vas_int_r), vastus lateralis(vas_lat_r), and vastus medialis (vas_med_r) muscles as a function of time over a gait cycle.

For the anatomically normal movement of the knee, active muscle forces are plotted with respect to time during gait as a baseline in order to have it compared against cases with different levels of impairments. The effect of the pin-jointed exoskeleton was observed through knee extensor and flexor muscle forces. Knee joint constraints were imposed based on two scenarios: (1) the pin-joint exoskeleton constrained the knee joint from moving along the x-direction (in sagittal plane), i.e., keeping the constant offset along the x direction (X = 0.00 mm); and (2) an imposed misaligned fit of the pin-joint to the biological joint by constant offsets of X = +5.00 mm, X = −3.67 mm and X = −6.40 mm in the anterior-posterior directions. Authors selected the scenarios based upon the range of knee joint displacements during gait (Figure 3). The scenarios are also compatible with the results shown in the anatomical knee joint (Figure 4).

### 2.1. Experimental Data

**Figure 5:**
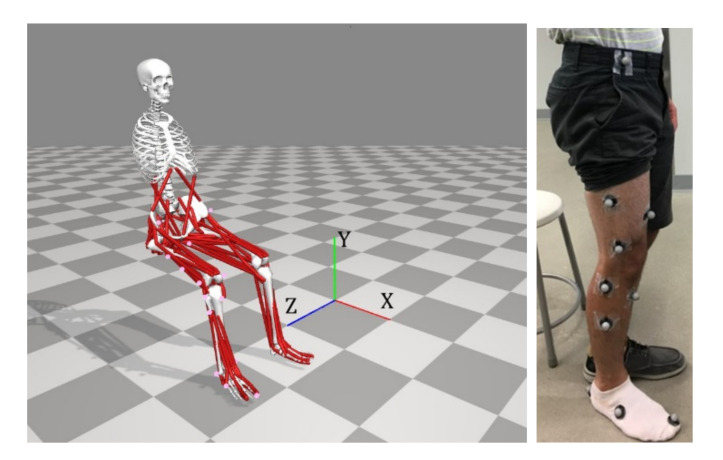
MoCap marker placement (**right**) and associated marker registration in OpenSim (**left**).

For the gait simulations, experimental gait data provided with the generic OpenSim gait models was used. This data consisted of a person 1.8 m tall with a mass of 75.16 kg walking on an instrumented treadmill at 1.15 m/s for 2.5 s [19]. Details on the right shank and foot clockwise rotation data collection are presented in detail elsewhere [4]. In summary, video motion capture data was obtained from a male subject seated with his hip and knee initially positioned at 90° angles in the sagittal plane. In order to capture the limb motion, 16 markers were placed on the subject’s thigh and shank, as shown in Figure 5. The thigh was unrestricted, and the foot was lifted off the ground prior to and during the circular motion.

### 2.2. Analysis of Motion Parameters Using Musculoskeletal Software Application

Video motion analysis marker coordinate data for both the gait and seated right shank and foot clockwise rotation motions were imported into OpenSim to calculate the kinematics of the individual body segments. The Inverse Kinematics (IK) tool was used to calculate a set of generalized coordinates that best represent the measured marker trajectories recorded in the experimental data. The IK tool solves a weighted least square problem (Equation (1)):

(1) $$\begin{array}{c} \min\\ q \end{array}[\overset{markers}{\underset{i=1}{\sum }}w_{i}{\left(x_{i}^{exp}-x_{i}\left(q\right)\right)}^{2}+\overset{u_{c}}{\underset{j=1}{\sum }}w_{i}{\left(q_{i}^{exp}-q_{i}\right)}^{2}].$$

where q indicates generalized coordinate vector, $x_{i}^{exp}$ signifies locations of markers in experiment i, $x_{i}\left(q\right)$ locates corresponding markers on the model, $u_{c}$ indicates the unspecified coordinates, and $q_{j}^{exp}$ shows actual value for coordinate j [18]. The IK tool was used with both unconstrained motions and constrained motions, for a total of four analyses. Once the IK analyses were performed, the Inverse Dynamics (ID) tool was utilized to compute the joint moments and reaction forces [20]. It provides generalized forces for all joints in a convenient way; however, there are some restrictions (e.g., uncertainties in estimating body segment parameters, force plate measurement accuracy, kinematic data processing, etc.) [21] and sources of error (e.g., anatomical landmark misplacement, skin motion, etc.) [22] involved in the process.

The Static Optimization (SO) tool in OpenSIM was used to calculate the muscle forces associated with the motions and constrained motions studied. The SO tool distributes overall joint moments into forces generated by each muscle at any time frame [18]. Basically, the movement of the model is equipped to solve the equations of motion for the forces under the following conditions (Equation (2)):

(2) $$\begin{array}{c} \overset{n_{i}}{\underset{i=1}{\sum }}\left(a_{i}F_{i}^{o}\right)r_{i,k}=\tau_{ki}\\ \overset{n_{i}}{\underset{i=1}{\sum }}\left[a_{i}f\left(F_{i}^{o},l_{i},v_{i}\right)\right]r_{i,k}=\tau_{kc}. \end{array}$$

as it tries to minimize the cost function (Equation (3)).

(3) $$J=\overset{n_{i}}{\underset{i=1}{\sum }}{\left(a_{i}\right)}^{p}$$

where $n_{i}$ represents the number of muscles consisted in the model, $a_{i}$ denotes the activation level of muscle i, $l_{i}$ denotes the muscle fiber length, $r_{i,k}$ denotes the moment arm of the muscle force about the *k*-th joint axis, $v_{i}$ denotes the shortening speed of the muscle, *p* denotes a constant defined by the muscle properties and $f\;\left(F_{i}^{o},\;l_{i},\;v_{i}\right)$ represents force-length-velocity criteria, $\tau_{ki}$ denotes the torque for ideal force generators, and $\tau_{kc}$ denotes the torque constrained by force-length parameters [23,24].

### 2.3. Knee Joint Assessment through Musculoskeletal Modeling and Simulation

To assess the effect of human–exoskeleton joint misalignment on the kinematics and the knee joint muscles of two motion configurations (i.e., gait and a prescribed circular motion of the shank and foot), simulations of movements unconstrained and constrained with different alignments and constraints imposed by a wearable exoskeleton were generated. The predefined gait motion files in the software associated with the OpenSim gait2392—simbody model and the trajectory of the end effector calculated in a previous work [4] were used as the baseline values for the unconstrained gait and circular motion of the shank and foot kinematics, respectively. The IK tool was used to calculate a trajectory of the right leg as it was expected to go through the path designated by the experimental motion capture marker set. Subsequently, knee joint muscle forces were calculated with the SO tool, based on the ID tool outputs. This process was repeated for each of the unconstrained and constrained motions.

**Figure 6:**
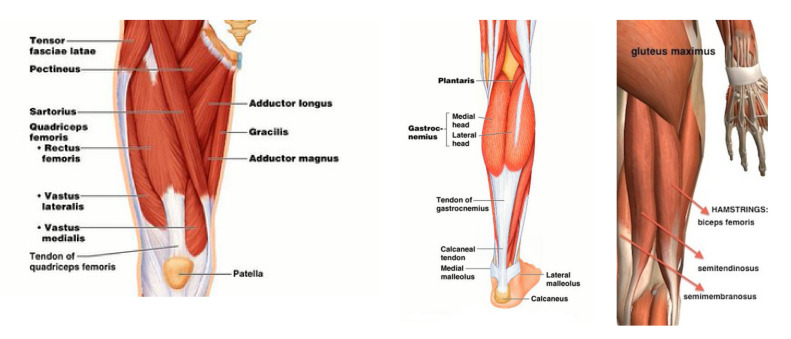
Anatomy of knee extensors and flexors (Pearson Edu.).

The key muscles for knee extension were the quadriceps encompassing the rectus femoris (RF), vastus lateralis (VL), vastus medialis (VM) and vastus intermedius (VI) muscles. The main knee flexors were the hamstrings, namely the semitendinosus (Semiten), semimembranosus (Semimem), biceps femoris short head (BFSH) and biceps femoris large head (BFLH) muscles. All eight knee-extensor and flexor muscles were incorporated in the leg model and investigated [25]. The gastrocnemius (Gastroc), sartorius (Sar) and gracilis (Grac) muscles were also observed for knee flexion and hip rotation for the circular motion of the shank and foot configuration, Figure 6. In all modeling processes, the weight of the exoskeleton weight was neglected. One of the limitations of the current study with regard to use of OpenSIM involves simplifications made to implement the effect of an exoskeleton in lieu of modeling an actual mechanism in the software. This approach will be considered in future studies as authors are currently working on continuation of the work. However, the current method is capable of implementing the required constraints intended for the study.

**Figure 7:**
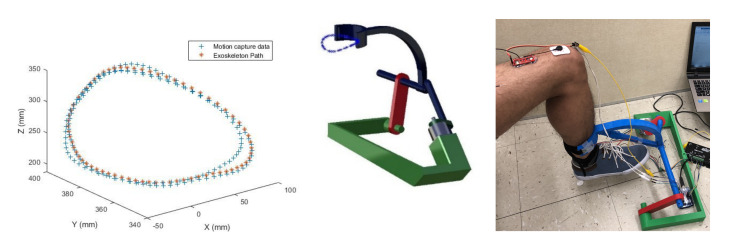
Trajectory generated by mechanism (blue) with the motion capture data (red); exoskeleton mechanism [4].

In the following, knee rotation in a seated position is then modeled and analyzed using MoCap data followed by investigating the effect of using an exoskeleton which was previously designed for a similar task by mechanism synthesis technique [4]. Based on the previous study [25], RF and VL show significant variations within muscles extending knee during gait. The same holds here, as the shank is moving through a closed circle-like loop (Figure 7). On the other hand, more flexing muscles are selected to be observed that will be further discussed in following sections.

**Figure 8:**
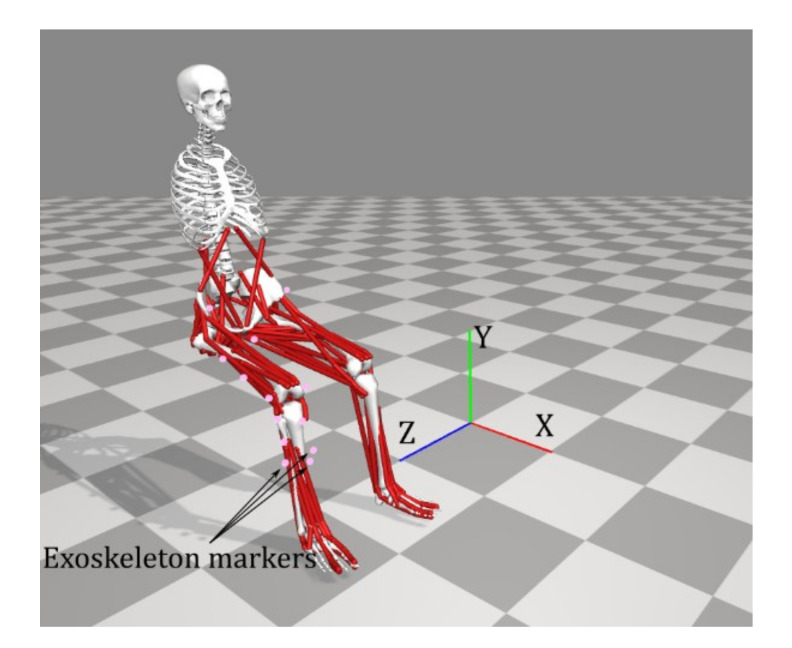
Marker registration for OpenSim model including three markers representing the exoskeleton seat.

As it was previously addressed, three markers indicating thigh movements along with three markers representing the exoskeleton end-effector are incorporated in the shank part (as shown in Figure 8) to model the knee rotation as it is constrained and rested on the exoskeleton fixture (Figure 7). This is intended to replicate an active exoskeleton that is driven by a motor and moves the shank segment of the foot through a circular trajectory in a transverse plane [4]. OpenSIM uses weight values for each marker and incorporates those values in IK equations; hence upon changing weight values pertinent to markers regarding the exoskeleton, trajectory of the shank movement would change accordingly which leads to different muscle force patterns. Therefore, this effect has been investigated in four cases where we gradually decrease the weight values related to the exoskeleton markers. To delineate, by having a very large value (i.e., 500) for three exoskeleton markers (Case I) we are ignoring all other markers except for those pertaining to the exoskeleton. This gives rise to an abnormally rigid movement where only the shank is moving in a stiff manner; the pelvis also shows slight movement in the transverse plane. On the contrary, using a very small weight value (i.e., 0) depicts a situation where no constraint is imposed from the exoskeleton to the leg (Case IV); however, in such cases, there would be 11 markers to describe the motion; meanwhile the model built from MoCap data had 16 markers that were used to introduce the lower extremity motion. The following explains the basis on which these four cases have been laid out: Eight markers are placed on the thigh and pelvis areas; two of which have been placed on two opposite sides of the knee (i.e., knee lateral, knee medial). In addition, three markers are placed on the thigh as well (Figure 8). These eleven markers have weight values of either 1 or 10. As for the first case, a weight value of 500 has been selected for three markers pertaining to the exoskeleton end effector. This is 50 times larger than the highest value within other markers. Subsequently, weight values of 200, 50 and 0 has been used as Case II, Case III and Case IV, respectively, to gradually impose an external constraint as the right knee tries to make a rotational movement in the transverse plane (X-Z in Figure 7 and Figure 8). Marker weight values are arbitrarily selected so as to introduce the exoskeleton effect. To better see the intensity of its impact, four weight values are chosen through four cases. The weight of the exoskeleton is neglected since the leg does not carry its load as shown in Figure 7.

## 3. Results and Discussion

### 3.1. Assessment of Flexion and Extension of the Knee Joint in Gait

**Figure 9:**
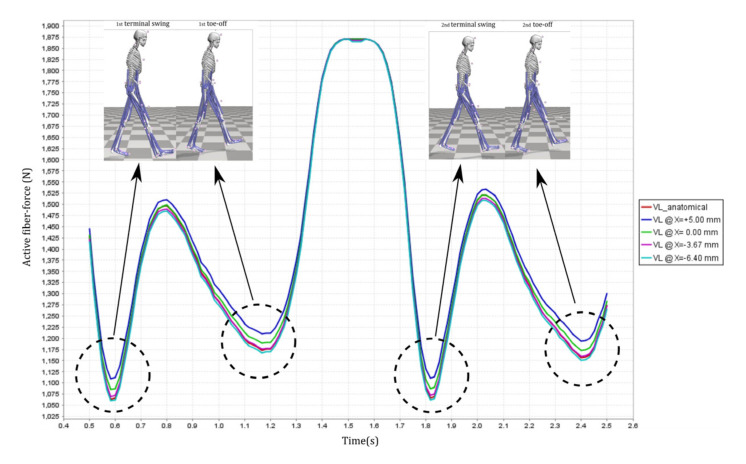
VL active fiber force for the anatomically healthy and various levels of impairment right foot.

Active fiber forces for knee extensors and flexors are shown in (Figure 4) providing a baseline for comparison with the four different levels of impairments. There were noticeable differences in the knee extensor and flexor muscle forces due to the effects of knee joint constraints and misalignment of the pin-jointed exoskeleton. For the cases in which the pin-joint exoskeleton has (1) constrained the knee joint from moving along the x-direction (i.e., keeping the knee joint aligned and at a constant X = 0.00 mm offset in the sagittal plane) and (2) imposed a misaligned fit between the exoskeleton pin-joint and the biological joint with constant offsets of X = +5.00 mm, X= −3.67 mm and X = −6.40 mm in the sagittal direction, the extensors exhibited more noticeable variations in force than the flexors. Among the knee flexors, there were negligible changes in the BFSH and BFLH forces. Moreover, Semiten and Semimem forces were almost unchanged across the range of exoskeleton misalignments; this implies insensitivity of the flexors to the level of knee joint misalignment. The stated changes will not introduce any clinical implications. Among the knee extensors, VL had the most pronounced variations that suggests as the level of misalignment increases, more stress will be delivered to the muscle (Figure 9). While RF force did not exhibit significant variations with the level of knee joint misalignment; VM and VI showed slight changes in generated muscle force.

As shown in Figure 9, the highest variability among the four imposed misalignments happens in the terminal swing and toe-off stages at a variability range of 50 N. Compared to the force variability throughout the entire gait, this counts for about 6.25% which is not problematic and is not going to cause any clinical implications. Moreover, the stated values for misalignment are numerically modeled; however, clinical measurements of such small deviations require an abundance of provisions.

### 3.2. Assessment of Training of the Knee with/without Synthesized Exoskeleton

The second scenario addresses analysis of knee rotation and also the implementation of the exoskeleton motion trajectory for rehabilitation purposes (Figure 2). In our previous work, an exoskeleton was designed following a circular trajectory of shank (Figure 7) while the subject was seated to recruit more muscles and expedite the rehabilitation process [4]. The mechanism is designed using real human kinematics data collected through MoCap. This novel exoskeleton was designed through mechanism synthesis, then 3D-printed, verified and assessed only based on its accuracy in mimicking the trajectory as shown in Figure 7. Here, we are demonstrating an additional assessment technique based on the comparison of the muscle forces generated while the shank is driven by those trajectories. This additional assessment will provide insight into whether the error in the trajectory can cause any complication. The same GAIT 23DOF 92MUSC model is used to simulate anatomical knee rotation by implementing MoCap marker set data. Here, one full cycle of rotation is modeled with a 0.5 s hesitation pause at the beginning. Figure 5 shows how markers are distributed with respect to the reference coordinate system.

Knee impairment due to joint or exoskeleton misalignment is investigated through flexion and extension of the knee in gait under different conditions; this is investigated in the first part of the study resulting in meaningful variations of the VL muscle which raises clinical concerns. While observing three cases where misalignments are implemented, reaction forces at the femur and tibia are explored. It was observed that such forces did not have a drastic change in the mediolateral direction (Z-direction). As for superior dimension (Y-direction), the right-leg stance phase showed the largest difference from the healthy condition. The anatomically healthy joint (X-direction) force trajectory is straddled by the two misalignment force trajectories suggesting that such exoskeleton misalignments could lead to increased shear stresses at the knee joint. Transverse stress is the main concern when patients suffer from possible pressure due to impairment or using misaligned exoskeletons.

**Figure 10:**
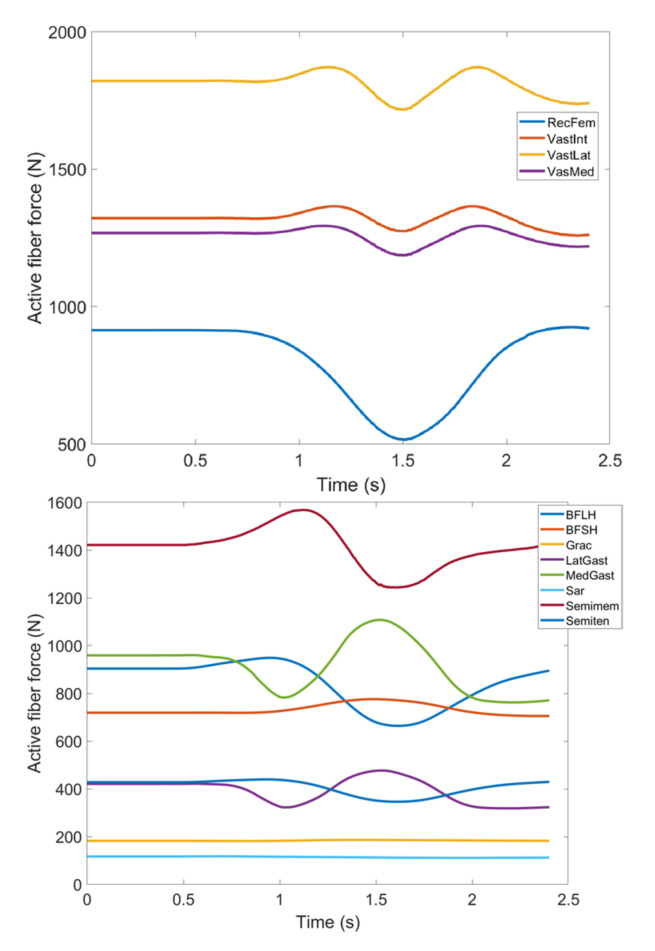
Active fiber force of muscles: Knee extensors (**Top**) and flexors (**Bottom**) regarding MoCap data (RecFem Rectus femoris, VastInt Vastus intermedius, VastLat Vastus lateralis, VasMed Vastus medialis, BFLH Biceps femoris large head, BFSH Biceps femoris short head, Grac Gracilis, LatGast Lateral Gastrocnemius, MedGast Medial Gastrocnemius, Sar Sartorius, Semimem Semimembranosus, Semiten Semitendinosus).

During knee movement, relatively large variations are also noticed with BFLH, Medial Gastrocnemius (MedGast) and Semimem muscles within knee flexors (Figure 10). This figure indicates major active fiber forces of main knee extensors and flexors as data has been collected through MoCap during knee movement.

**Figure 11:**
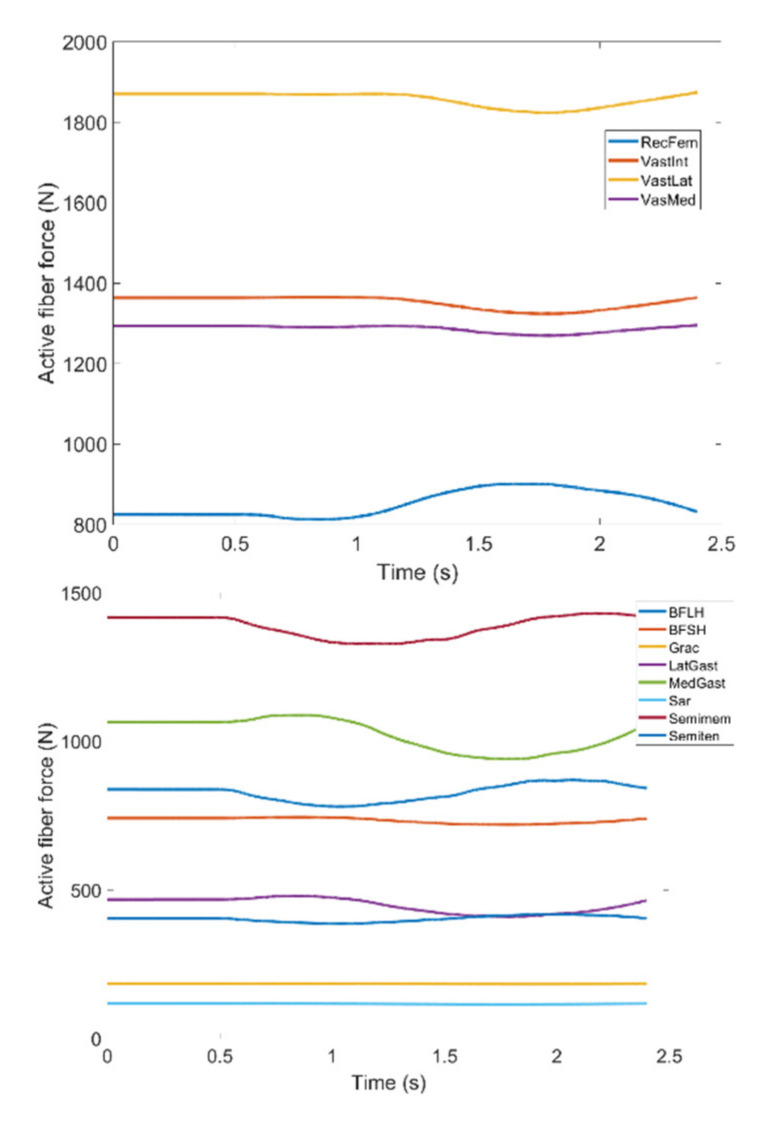
Case I: Active fiber force of muscles: Knee extensors (**Top**) and flexors (**Bottom**).

**Figure 12:**
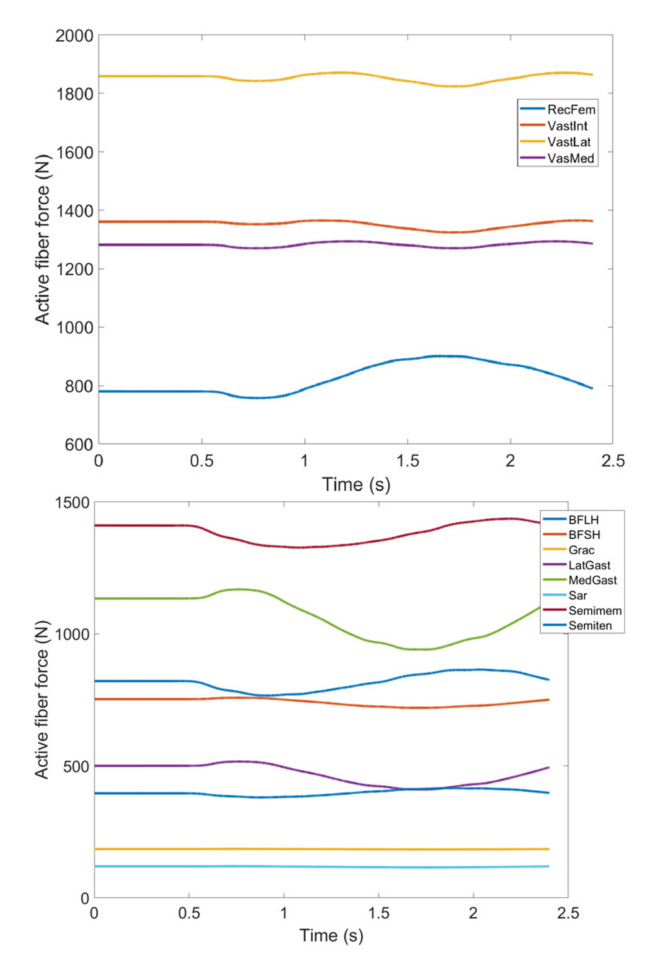
Case II: Active fiber force of muscles: Knee extensors (**Top**) and flexors (**Bottom**). Knee extensors and flexors are listed in Table 2.

**Figure 13:**
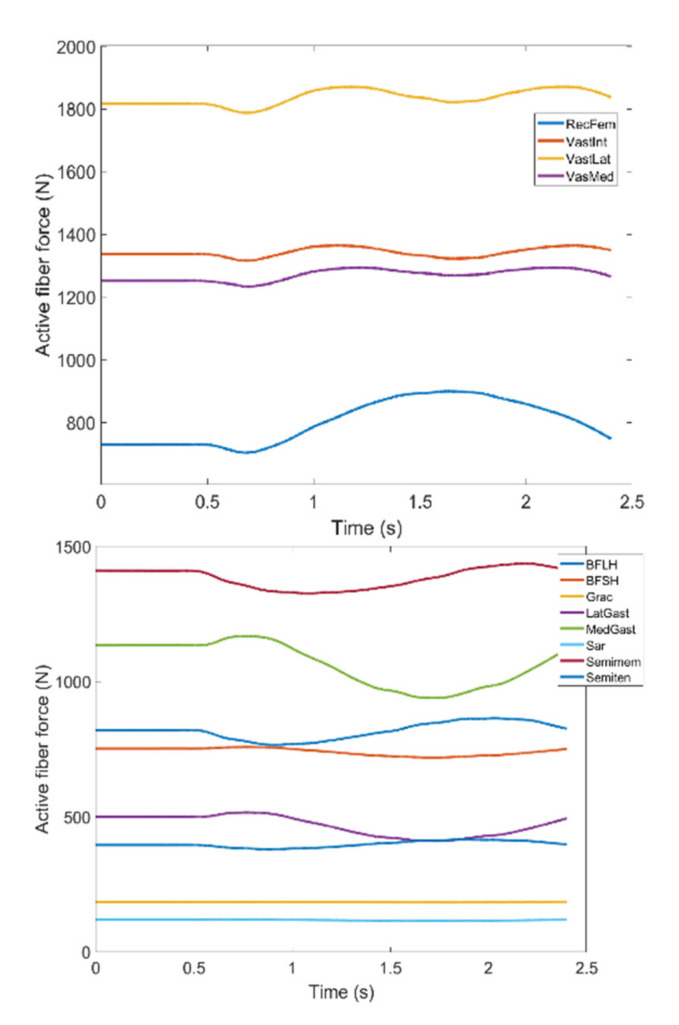
Case III: Active fiber force of muscles: Knee extensors (**Top**) and flexors (**Bottom**). Knee extensors and flexors are listed in Table 2.

**Figure 14:**
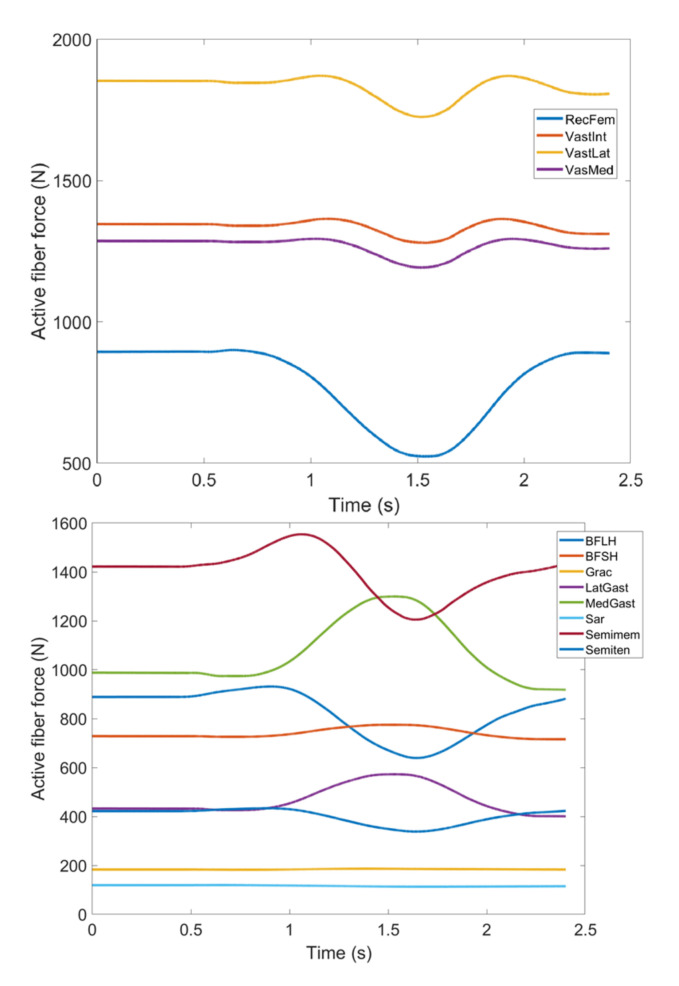
Case IV: Active fiber force of muscles: Knee extensors (**Top**) and flexors (**Bottom**). Knee extensors and flexors are listed in Table 2 (RecFem Rectus femoris, VastInt Vastus intermedius, VastLat Vastus lateralis, VasMed Vastus medialis, BFLH Biceps femoris large head, BFSH Biceps femoris short head, Grac Gracilis, LatGast Lateral Gastrocnemius, MedGast Medial Gastrocnemius, Sar Sartorius, Semimem Semimembranosus, Semiten Semitendinosus).

As stated earlier, weight values of 200, 50 and 0 were used as Case II, Case III and Case IV, respectively, to gradually impose an external constraint as the right knee tries to make a rotational movement in the transverse plane (X-Z in Figure 8); associated muscle forces for main extensors and flexors are demonstrated in Figure 11, Figure 12, Figure 13 and Figure 14. The first half a second represents a short pause followed by one cycle of rotation which takes 1.9 s. According to the graphs regarding Case I to Case IV, it is evident that RF and VL muscles show higher fluctuations through the movement time within extensors; as for flexing muscles, MedGast, Semimem and BFLH are demonstrating major variations. This is in accordance with what was indicated by the MoCap data in Figure 10. In order to better investigate the impact of the constraint due to the exoskeleton on each muscle, normalized muscle forces are calculated and plotted. This helps with a better understanding of the significance of variations since muscle force magnitude is divided by the maximum force of the pertinent muscle. OpenSIM plots were not smooth, hence a robust local regression filter is also applied to deliver smoother graphs. It is noteworthy that data regarding Case IV can also be implied as verification of the MoCap analysis except with smaller numbers of markers.

(1) **Extensors:** As expected, muscle forces stayed consistent in the hesitation phase (0–0.5 s). As described earlier, the first three cases were meant to demonstrate the incremental activation of the exoskeleton constraint (i.e., Case I being exoskeleton markers heavily dominating the movement path, and Case III being the markers contributing to the movement) and last two being discarding exoskeleton markers (Case IV) and MoCap data (Case V) are rather close which agrees with the intent. It should be noted that discrepancies between Case IV and Case V are possibly due to different numbers of markers used to produce the associated motion.

**Figure 15:**
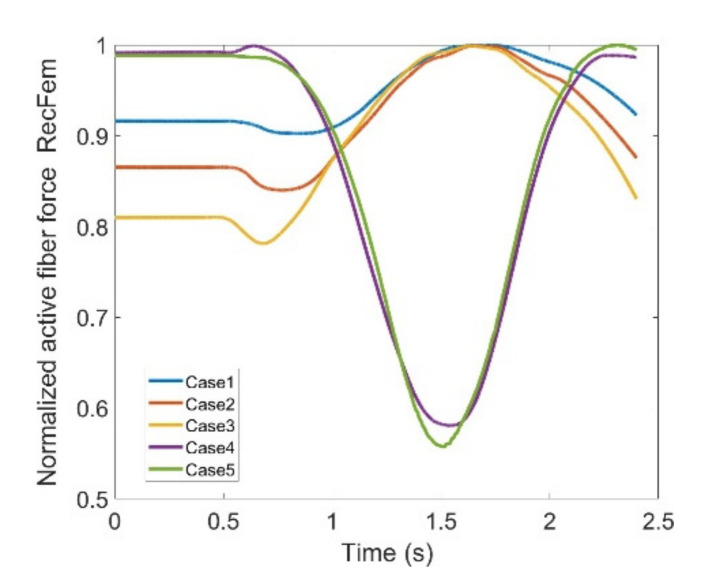
Normalized active fiber force of the Rectus Femoris (RF) muscle for different cases, Case5 being MoCap.

In observing RF, there is not a significant difference between the two. Given that, the first three cases can be fairly compared with the last two as the two groups drastically differ in pattern. Based on Figure 15, the difference reaches as high as 44% of the maximum fiber force in RF muscle within five defined cases. This is the biggest difference among other main knee extensors and considering high values for active fiber forces regarding this muscle (Figure 15) could be problematic by imposing extra pressure in case the exoskeleton is used.

**Figure 16:**
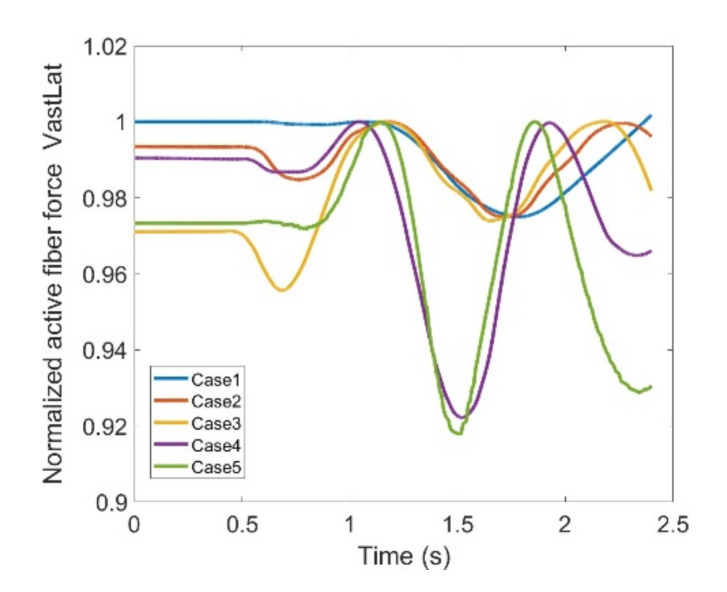
Normalize active fiber force of the Vastus Lateralis (VL) muscle for different cases, Case5 being MoCap.

**Figure 17:**
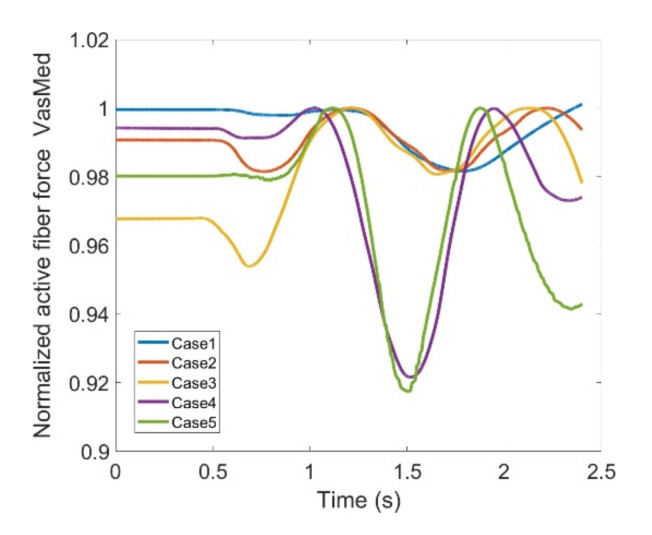
Normalized active fiber force of the Vastus Medialis (VM) muscle for different cases, Case5 being MoCap.

**Figure 18:**
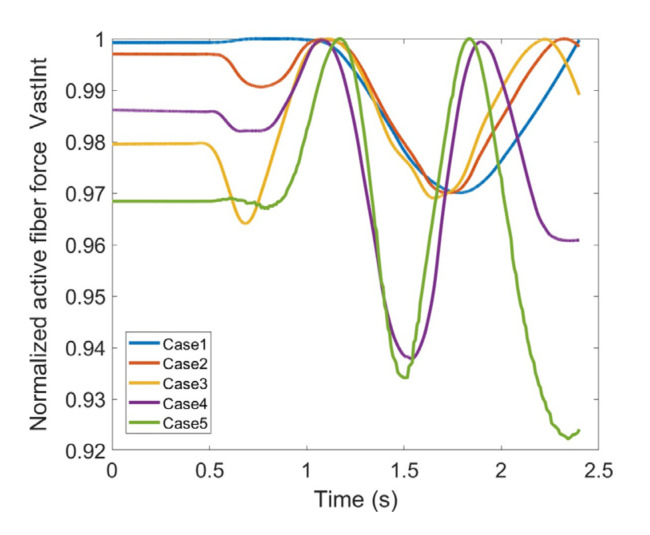
Normalized active fiber force of the Vastus Intermedius (VI) muscle for different cases, Case5 being MoCap.

VL, VM and VI have also been investigated through Figure 16, Figure 17 and Figure 18; all graphs indicate the last two cases have offset at the hesitation phase, but their trends become similar as the knee starts to move. Again, there are distinguishable differences between the first three and last two cases which enables us to compare the unconstrained movement of the knee against the knee moving by the exoskeleton toward the rehabilitation process. There are noticeable peaks and drops in all three graphs, but the variation reaches to 8% of the maximum muscle fiber force at the highest. Although the generic pattern is similar within the three muscles, VL has a relatively higher force fiber magnitude compared to VM and VI. Despite that, 8% variation is considered to be within a safe margin and none of these three muscles impose discomfort in case the exoskeleton is utilized.

(2) **Flexors:** The same analysis is conducted for major knee flexors; and active fiber forces regarding these muscles are compared in different scenarios among various cases. Hamstrings are a group of muscles encompassing the Biceps Femoris Large Head (BFLH), BFSL, Semimem; and Semiten that are first observed within flexors.

**Figure 19:**
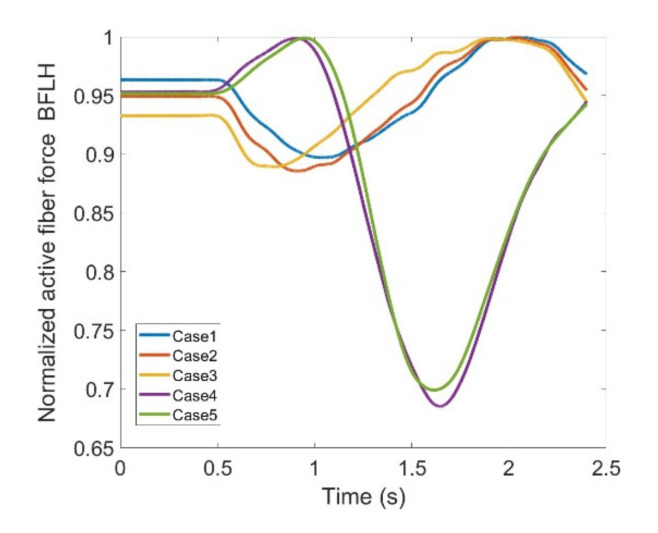
Normalized active fiber force of the BicepsFemoris LargeHead (BFLH) muscle for different cases, Case5 being MoCap.

**Figure 20:**
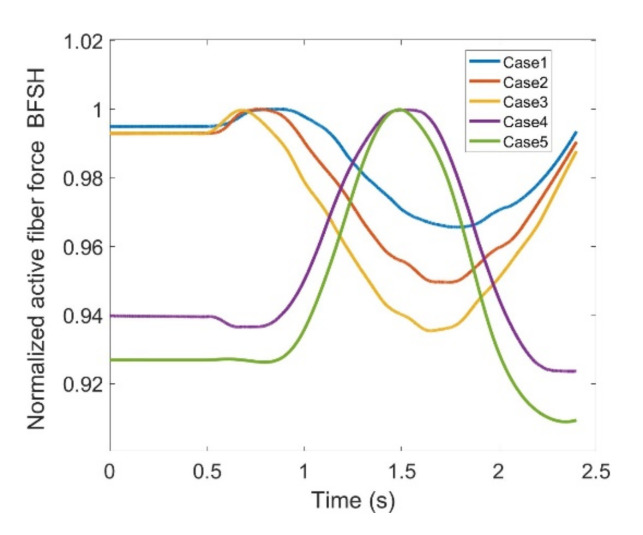
Normalized active fiber force of the BicepsFemoris ShortHead (BFSH) muscle for different cases, Case5 being MoCap.

Muscle forces for the BFLH are shown in Figure 19 where there is a fairly good correlation between the anatomical knee movement and the modeled one (Case V vs. Case IV). There are also peaks and drops in both patterns and it could be deduced that changes may reach as high as 32% making this BFLH muscle somewhat questionable in terms of imposed complications attributed to using the exoskeleton. BFSH, however, has shown 9% variation at the most which implies that the muscle is not affected while the knee is being trained by the exoskeleton (Figure 20).

**Figure 21:**
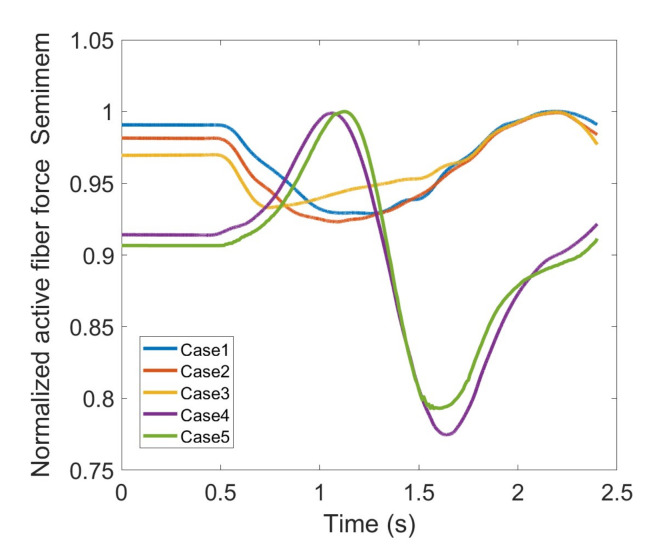
Normalized active fiber force of the Semimembranosus (Semimem) muscle for different cases, Case5 being MoCap.

**Figure 22:**
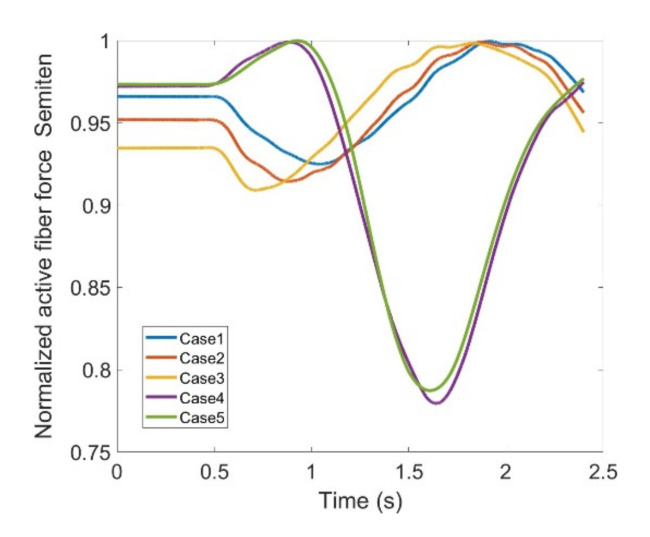
Normalized active fiber force of the Semitendinosus (Semiten) muscle for different cases, Case5 being MoCap.

Figure 21 and Figure 22, Case IV seems to be well aligned with the anatomical knee rotation processed by MoCap data set. The small deviation can be interpreted toward model verification with lower number of markers (Case IV), yet both muscles experience a peak and a drop; the largest muscle force difference could reach 23% and 22% of the maximum active muscle fiber force for Semimem and Semiten, respectively. Even though, based on the normalized graphs, Semimem and Semiten have demonstrated almost equivalent variations accompanied by similar trends, Semimem force is at a significantly higher level than Semiten making it more essential to screen when utilizing the exoskeleton for training purposes.

**Figure 23:**
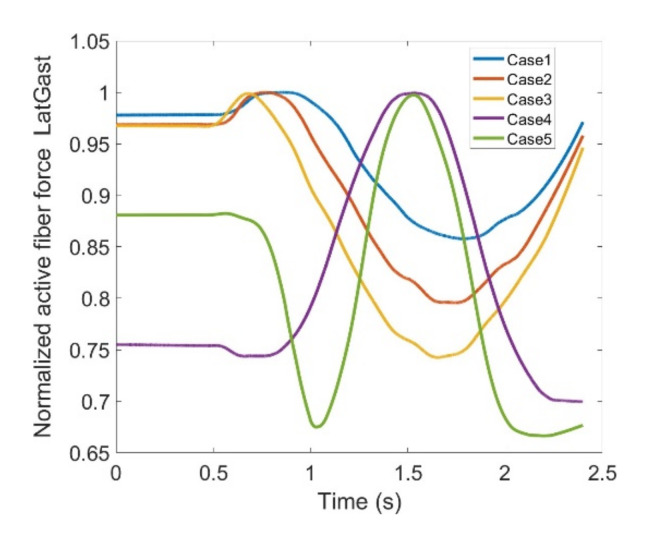
Normalized active fiber force of the Lateral Gastroc (Lat Gas) muscle for different cases, Case5 being MoCap.

**Figure 24:**
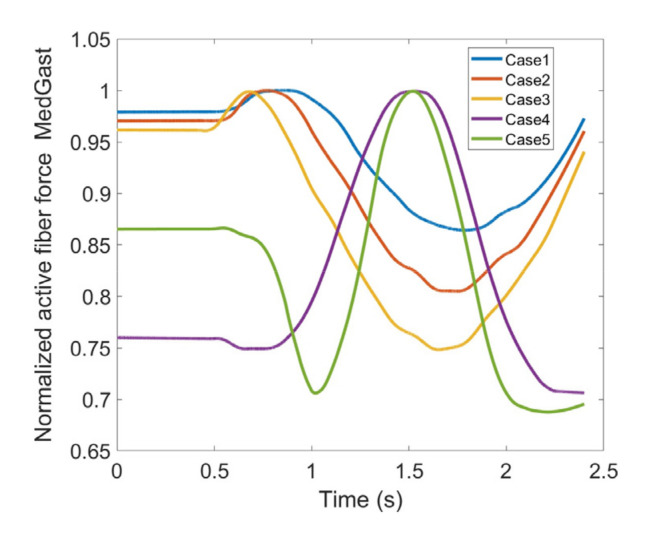
Normalized active fiber force of the Medial Gastroc (MedGas) muscle for different cases, Case5 being MoCap.

In addition to hamstrings, Gastroc which is a two headed muscle (Figure 6) also participates in flexion of the knee joint. In Figure 23 and Figure 24 Lateral (LatGas) and Medial Gastroc (MedGas) muscles are observed. Case IV and V have shown offsets which might be due to a reduced number of markers to define movement in Case IV compared to the anatomical one (Case V); though their patterns are yet comparable. MedGas reveals significant contribution toward knee flexion as Figure 24 reflects larger muscle force. Based on Figure 23 and Figure 24, the force variations between different cases lie within the range of 33% and 31% of the maximum force for LatGas and MedGas, respectively. Given these discrepancies, MedGas seems to be more crucial as it carries a larger load in knee flexion. Given that, both muscles could be counted critical to monitor as they endure rather large variations as a consequence of using the exoskeleton.

**Figure 25:**
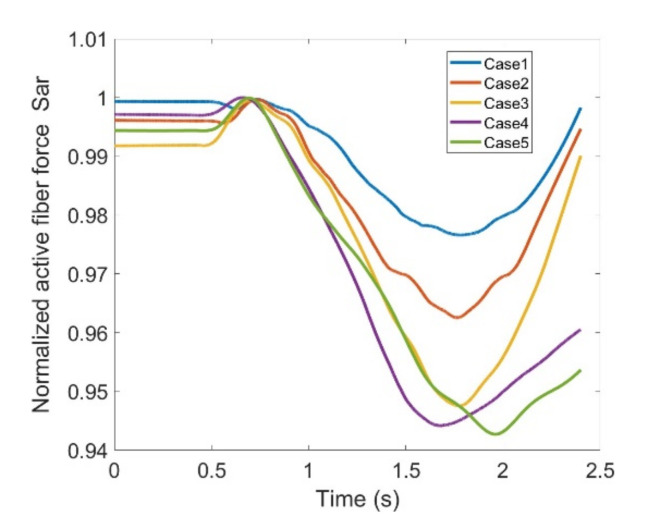
Normalized active fiber force of the Sartorius (Sar) muscle for different cases, Case5 being MoCap.

**Figure 26:**
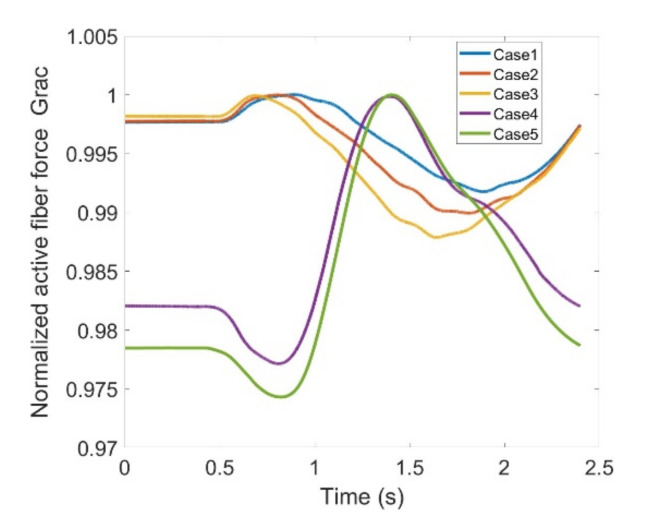
Normalized active fiber force of the Gracilis (Grac) muscle for different cases, Case5 being MoCap.

In the end, Sar and Grac muscles are assessed through Figure 25 and Figure 26 indicating fiber forces as well as normalized forces based on the maximum fiber force of each pertinent case. Sar deals with abduction of the thigh at the hip joint and also flexes the knee joint. When using knee joint training with an exoskeleton it is apparent that Sar does not contribute too much to carry load as the fiber force trend does not show remarkable changes within the five defined cases here in this study. Moreover, variation range peaks where it reaches 3% and force level is rather low compared to other flexors. The same is true for Grac. It indicates 6% of variation range with relatively lower force level. Grac helps with thigh adduction as well as knee flexion; although there exist discrepancies where the exoskeleton is utilized but its magnitude is relatively low for both muscles. This implies that Sar and Grac flexors will not be heavily impacted or cause any complications during rehabilitation. Table 2 summarizes the variation ranges regarding each contribution muscle. It has been deduced that RF, Gastro, BFLH, Semimem and Semiten muscles need extra attention when this particular exoskeleton is equipped in order to provide assistance in rehabilitation of the knee.

In summary, Shear stresses were assessed at the femur and tibia interface to better understand the nature of pressure due to a misaligned exoskeleton where transverse stress on the knee joint force reaction on the tibia was found meaningfully different in posterior direction (X-dim) during gait. Such considerations need to be taken into account when knee training efficacy is assessed.

This was followed by an assessment of training of the knee joint without and with a novel exoskeleton that was designed through mechanism synthesis and then 3D printed. This knee rehabilitation mechanism is designed using real human kinematics data collected through MoCap as the subject was moving his shank through a circular path while seated. Exact kinematics synthesis was performed to ensure smoothness of motion between task positions while generating the desired path and providing support to the shank

When comparing actual human movement with associated simulation, there are limitations involved. The actual human gait may differ from the outcome of this study and adaptability of the model may depend on several factors such as anthropometric characteristics of subjects, history of injury or stroke, weight and so on. Other limitations of the current study with regard to use of OpenSIM are simplifications made to implement the effect of an exoskeleton in lieu of modeling an actual mechanism in the software. Based on the shortcomings of conventional physiotherapeutic tasks, qualitative outcomes of this study can be helpful in prescribing an impactful yet convenient configuration toward a safe and promising rehabilitation process.

## 4. Conclusions

In this study, a constraint knee joint in a misaligned exoskeleton was investigated in gait. Four levels of misalignments were designated to examine knee flexors and extensors; consequently, the Vastus lateralis muscle showed the most noticeable variations that can potentially impose undesirable pressure on the knee as the right leg was at the terminal swing and toe-off phase. That is up to 4.3% due to a 5 mm lateral offset from an anatomically healthy knee joint location.

As the joints of the exoskeleton mechanism are oriented/located independent of the anatomical landmarks of the knee joint; this will reduce the joint alignment and stress on the user. Among knee extensors, the Rectus Femoris showed a remarkable variation of 44%. As for flexors, BFLH and Gastroc muscles exhibited noticeable variations (32% and 33%, respectively) arising the need for extra caution when using the trajectory during rehabilitation. Such meaningful differences in normalized muscle forces are proper measures to monitor knee rehabilitation efficacy especially when physicians are interested in targeted muscles training. Additionally, such significant changes may lead to clinical implications. This profusely happens in the rehabilitation of elite athletes when a certain muscle needs to be probed.

Results from this pilot study will benefit the design of experiments in future studies as authors are currently continuing towards expansion of the work. In future studies, sEMG data can be incorporated to furnish better understanding of muscle forces and to confirm when forces increase, that coincides with a spike in the associated sEMG signal. This indicates that the muscle is active and force is generated through fibers. sEMG analysis can be accompanied by a large number of the patient population and pertinent statistical analysis.

## Figures and Tables

**Table 1:** Related studies.

Authors	Objective	Findings	Significance	Limitations
Bessler-Etten, et al. [13]	Investigating the effects of misalignment in different directions and rotationally to find if there is an increased load on the knee joint.	Misalignment caused an increased load on the knee joint in both translation and rotation.	First to look at the relative effect of different amounts and directions of misalignments.	The study was conducted using physical models that could not assess the individual muscle force outputs.
Sarkisian, et al. [14]	Determine if the Utah Exo Knee self-aligning mechanism improves user comfort and efficacy.	The Utah ExoKnee self-aligning mechanism can improve user comfort and efficacy in high-torque, high-power and low-torque, low-power applications.	The first to assess user comfort and performance when using a self-aligning mechanism with a powered knee exoskeleton.	Only to be used in cases involving the knee joint. Forces in the Y-axis and X-axis were not reduced from a passive degree of freedom.
Sarkisian, et al. [15]	Investigate whether rotational misalignments from the Utah Exo Knee exoskeleton causes an increase in spurious forces and torques on the joint.	The Utah ExoKnee alignment did not affect the torque or spurious interaction forces. The self-aligning mechanism compensated for misalignments in high-torque and high-power applications.	The first to experimentally validate the torques and interaction forces using direct measurements. Used passive degrees of freedom to limit misalignment that could occur.	Did not consider user comfort. Required manual adjusting per participant, where having an automatic system would be preferred.
Cevik, et al. [16]	Design a lower limb brace for exoskeletons to reduce interaction forces caused by misalignment.	The designed brace allowed for forces in the X-axis and Y-axis. The forces in the Z-axis could not be reduced since the weight acts through the Z-axis.	Took a new look at how to compensate for misalignments by using passive degrees of freedom and by locking certain joints.	Specialized to a certain exoskeleton model. Was not able to connect to the shank. It was not assessed for comfort.
Li, et al. [17]	Create a modeling framework that is capable of understanding the interaction forces between the knee joint and an exoskeleton.	The framework was able to model the human knee joint during gait cycles.	Developed an independent modeling system to assess the interaction forces between the knee joint and an exoskeleton.	Only to be used on the knee joint and in modeling of exoskeletons.

**Table 2:** Comparison of variation range for knee extensors and flexors.

**Knee Extensor**	**Differential Range (Normalized)**
Rectus Femoris (RF)	44%
Vastus Intermedius (VI)	8%
Vastus Lateralis (VL)	8%
Vastus Medialis (VM)	8%
**Knee Flexor**	**Differential Range (Normalized)**
Biceps Femoris-long head (BFLH)	32%
Biceps Femoris-short head (BFSH)	9%
Gracilis (Grac)	3%
Lateral Gastrocnemius (LatGast)	33%
Medial Gastrocnemius (MedGast)	31%
Sartorius (Sar)	6%
Semimembranosus (Semimem)	23%
Semitendinosus (Semiten)	22%

## Data Availability

Not applicable.
